# Prediction of Small Bowel Obstruction Caused by Bezoars Using Risk Factor Categories on Multidetector Computed Tomographic Findings

**DOI:** 10.1155/2016/6569103

**Published:** 2016-06-15

**Authors:** Lian-qin Kuang, Da-wei Zhao, Cheng Cheng, Yi Wang

**Affiliations:** Department of Radiology, Institute of Surgery Research, Daping Hospital, Third Military Medical University, Chongqing 400042, China

## Abstract

*Objectives*. The aim of this study was to detect factors associated with small bowel obstruction (SBO) caused by bezoars on multidetector computed tomographic findings.* Methods*. We retrospectively reviewed 61 patients who had bezoars in the small bowels on MDCT. The patients were divided into SBO patients group and non-SBO patients group. The mean values of the diameter, volume, and CT attenuation as well as location and characteristics of the bezoars were compared between the two groups. Multivariate analysis was performed to determine factors associated with SBO.* Results*. There were 32 patients (52.5%) in the SBO group and 29 patients (47.5%) in the non-SBO group. The bezoars in the SBO group had greater values of each mean diameter and mean volume than those in the non-SBO group (3.2 ± 0.5 cm versus 1.6 ± 0.7 cm, *P* < 0.0001, 14.9 ± 6.4 cm^3^ versus 2.5 ± 2.7 cm^3^, *P* < 0.0001, resp.) and had a lower CT attenuation than the non-SBO group (55.5 ± 23.4 versus 173.0 ± 68.0, *P* < 0.0001). The SBO group had higher prevalence of phytobezoar appearance (75.0% versus 10.3%, *P* < 0.0001). Major diameters of bezoar and phytobezoar were significant independent risk factors associated with SBO (odds ratio = 36.09, 8.26, resp., and *P* = 0.0004, 0.044, resp.).* Conclusions*. Major diameter of bezoar or phytobezoar is a potential risk factor associated with SBO.

## 1. Introduction

In 2009, multidetector computed tomography (MDCT) has been reported to be useful in the diagnosis of small bowel obstruction (SBO) caused by bezoars. MDCT images reveal typical appearances in both gastric and small bowel bezoars and improve diagnostic accuracy [1]. However, published literatures on MDCT findings on a large scale involving different types of bezoars causing SBO are limited except for scattered case reports [2–14] and a few retrospectively comparative studies [15–18].

The possibility of bezoars eventually resulting in SBO might be associated with their size, location, type, and other factors. However, so far, to the best of our knowledge, there is yet a lack of systematic research evaluating the relationships between these factors of small bowel bezoars and the associated risk of causing SBO. It is therefore necessary to explore the risk factors of SBO resulting from small bowel bezoars.

MDCT with multiple postprocessing reformations is used as an investigative tool to study the types, size, locations, and other factors of small bowel bezoars. These factors are easily determined from their features observed on multiple reformation images acquired from MDCT scans. The purpose of this study was to perform a systematic evaluation of the MDCT features of small bowel bezoars, with and without SBO, and to identify the risk factors resulting in SBO in patients with small bowel bezoars. Although bezoar in small bowel is rare disorder, it potentially causes SBO. This article describing SBO caused by bezoar will contribute to emergency radiology.

## 2. Materials and Methods

### 2.1. Study Subjects

This study was approved by the institutional review board of our hospital. The requirement for written informed consent was waived for this retrospective study. A computerized search of clinical databases from our institution between January 2006 and December 2014 revealed 61 patients with small bowel bezoars diagnosed by using MDCT. The clinical and radiological data of these patients were reviewed. With radiological diagnostic criteria, these patients were divided into two groups, the SBO group (32 patients) and non-SBO group (29 patients), based on the presence of bezoars, with or without SBO. Patient's backgrounds were compared between the two groups with regard to the mean values of age, body mass index (BMI), and male/female ratio. All 61 patients underwent an abdominal MDCT and abdominal radiograph, and 9 of them underwent gastrointestinal contrast radiography within 3 days before the treatment.

### 2.2. Radiographic Examinations

Abdominal radiograph was acquired by using a high-frequency X-ray photography unit (AXGP20, Siemens AG, Munich, Germany) at 70–80 kV and 20–30 mAs, with the patient in a standard upright and supine position. Gastrointestinal tract contrast studies were performed on digital fluoroscopy equipment (VS–20D, Shimadzu Co., Kyoto, Japan). Each patient had a fasting time of 10–12 hours prior to examination. The patients were orally administered 80–100 mL of a 65% diatrizoate meglumine sodium (Angiografin, Belimed, Shanghai, China) and 20 mg metoclopramide (Otsuka Pharmaceutical Co., Ltd., Chengdu, China). Video fluoroscopy was performed during the contrast agent which was administration.

### 2.3. MDCT Examinations

All of the patients that we reviewed underwent unenhanced and dynamically enhanced (hepatic arterial, portal venous, and equilibrium phases) CT scanning from the diaphragm to the symphysis pubis on 64-row MDCT scanner (LightSpeed VCT, GE Healthcare, Milwaukee, USA). Patients did not receive any oral contrast agent prior to the routine scan, but four of them underwent gastrointestinal contrast radiography within 48 hours prior to the CT scanning. For enhanced scanning, a dual-head power injector was used to administer a flush of Iopromide (Ultravist; Bayer Schering Pharma, Berlin, Germany) at 370 mg iodine/mL followed by 30 mL saline. The contrast agent and saline solution were injected at 4 mL/s through an 18-gauge plastic intravenous catheter placed in an antecubital vein. Contrast agent volumes were delivered at 2 mL/kg body weight, and the upper limit of dose was set to 120 mL for every patient.

The scanning parameters were a detector configuration of 64 × 0.625 mm, slice thickness and reconstruction interval 0.625, table speed 64 mm per rotation, pitch 0.984, matrix 512 × 512, field of view 180–240 mm, tube voltage 120 kV, and tube current 300 mA. Images were routinely reformatted with a slice thickness of 5 mm and 3 mm at the position of transverse and coronal plane, respectively, by using an off-line workstation (ADW4.3; General Electric Healthcare, Milwaukee, USA). The source images were postprocessed by using volume rendering (VR), multiplanar reformation (MPR), maximum intensity projection (MIP), and curved planar reformation (CPR) for bowel segments where the bezoars were located.

### 2.4. Image Analysis

Two certified radiologists with 8 years and 10 years of experience in abdominal MDCT analyzed the bezoar features with regard to the location, type, number, size, and shape. Secondary changes were evaluated for the presence or absence of bowel dilatation and wall thickening, target sign, and the involved bowel segment and mesentery. The ancillary findings in the peritoneal cavity were also analyzed. In cases of discrepancies in the interpretations of the two radiologists, a consensus was reached.

The location of the bezoars was categorized as proximal (duodenum to proximal jejunum), middle (mid-jejunum to mid-ileum), distal (distal ileum), or multisegmental (involving more than one segment) according to modified Cole's method [19]. The node or mass was classified as phytobezoar appearance: a round, ovoid, or tubular, well-defined, heterogeneous, intraluminal mass with a mottled air pattern [2–5, 16, 21]; calcareous bezoar appearance: a well-defined, round or ovoid, intraluminal hyper dense nodule [20]; and trichobezoar appearance: a large, well-circumscribed, nonhomogeneous lesion in the lumen of the stomach with a tail extending up to the jejunum or beyond, which was composed of concentric whorls of different densities comprising pockets of air enmeshed within it [21, 22]. Maximum length and width of the bezoar were measured on the transverse images and maximum craniocaudal depth was measured on the coronal images. Volumes were calculated by using the following ellipsoid formula: length × width × depth × 0.5233.

The degree of obstruction on the basis of small bowel distension was graded as no (<2.5 cm), mild (2.5–2.9 cm), moderate (3-4 cm), or severe (>4 cm). Bowel wall enhancement was calculated as the absolute difference in the attenuation value of the bowel wall between the unenhanced and contrast-enhanced images obtained during the arterial and portal phases. Contrast enhancement was defined as increased attenuation of > 20 HU. Mesenteric changes included vascular engorgement and haziness. Vascular engorgement was indicated when there was an increase in the number and the size of the mesenteric vessels was increased, and the presence of mesenteric haziness was suggested when the attenuation of the mesenteric fat was increased.

### 2.5. Statistical Analysis

Results are presented as means ± SDs. Statistical analysis was performed using the SPSS 18.0 statistical software package (SPSS Inc., Chicago, USA). Continuous variables were tested for normality and equality of variances using the Kolmogorov-Smirnov test and the Levene *F*-test, respectively. Student's *t*-test, Pearson's chi-squared test, and Fisher's exact test were used when appropriate. A *P* value of less than 0.05 was considered significant. Logistic regression analysis (multivariate analysis) should be performed to know the factors associated with SBO. Variables significant at *P* < 0.05 and *B* (regression coefficient) > 0 by univariate analysis were subjected to forward stepwise logistic regression analysis to identify independent risk factors for SBO.

## 3. Results

### 3.1. Clinical Characteristics

The differences in the clinical characteristics between the two groups were not significant (*P* > 0.05) (Table 1). In the SBO group, all patients had abdominal pain, 15 had abdominal distension, 13 had constipation, 10 had nausea and vomiting, and 4 had an abdominal mass. In the non-SBO group, 26 had abdominal pain, 3 had melena, and 2 patients received an examination due to other causes. The duration of symptoms in all the patients admitted to the hospital ranged from one day to one month, with an average of 11.88 ± 7.61 days and 7.76 ± 6.83 days for the SBO group and non-SBO group, respectively. The differences in abdominal pains, abdominal mass, and melena between the two groups were not significant (*P* > 0.05). However, none of the non-SBO patients had abdominal distension, constipation, nausea, or vomiting. The SBO group had a longer mean duration of symptom before admission than those in the non-SBO group with a significant difference (*P* = 0.031) (Table 1).

### 3.2. Radiographic Findings

Plain abdominal radiograph demonstrated various grades of dilated loops in the small bowel suggesting SBO in the obstructive group. Of the 32 patients with SBO, 18 patients had moderate to severe grade SBO, 12 had mild grade SBO, and 9 had complete SBO confirmed by digital fluoroscopy. Other positive findings included the presence of gallstones in 2 patients and kidney stones in 1 patient. In the non-SBO group, there was no positive sign of SBO except for the presence of kidney stones in 2 patients observed on plain abdominal radiograph. All 29 patients without SBO did not undergo further gastrointestinal tract contrast studies.

### 3.3. MDCT Findings

**Figure 1:**
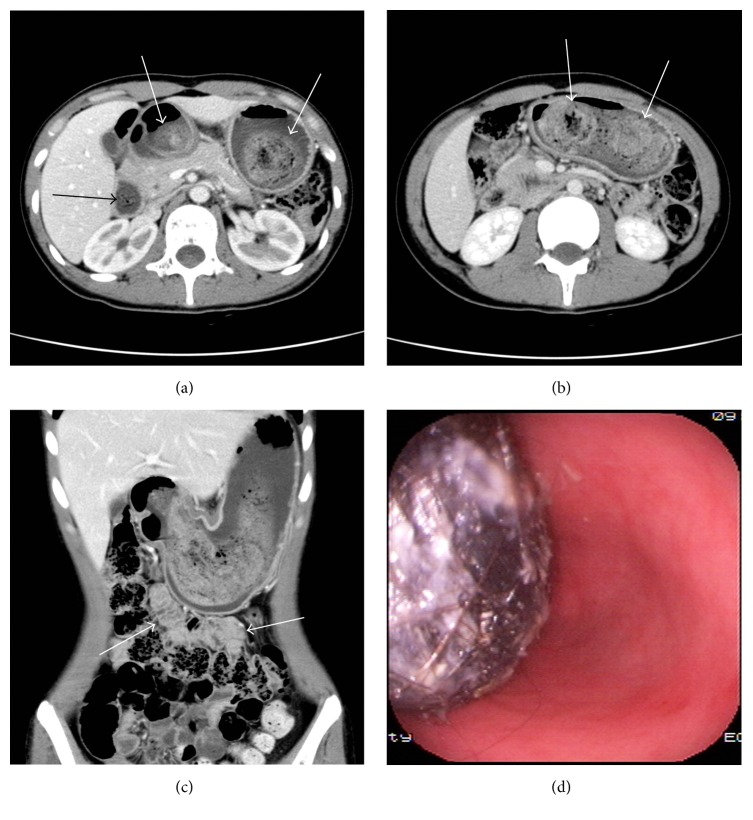
Trichobezoar in the gastrointestinal tract causing high-level intestinal obstruction. (a, b) MDCT axial images of the upper-middle part of the abdomen demonstrated a large, well-circumscribed, nonhomogeneous lesion (white arrows) in the lumen of the stomach with a tail extending up to the duodenum (black arrow) that was composed of concentric whorls of different densities. This suggests a positive diagnosis for trichobezoar. (c) MDCT coronal image shows the trichobezoar in the stomach cavity extending up to proximal jejunum (arrows). (d) Gastroscopy and surgical operations detected a giant trichobezoar filling about 75% of the stomach cavity, extending through the pylorus and duodenum into proximal jejunum. This is covered with viscous liquid secretions.

**Figure 2:**
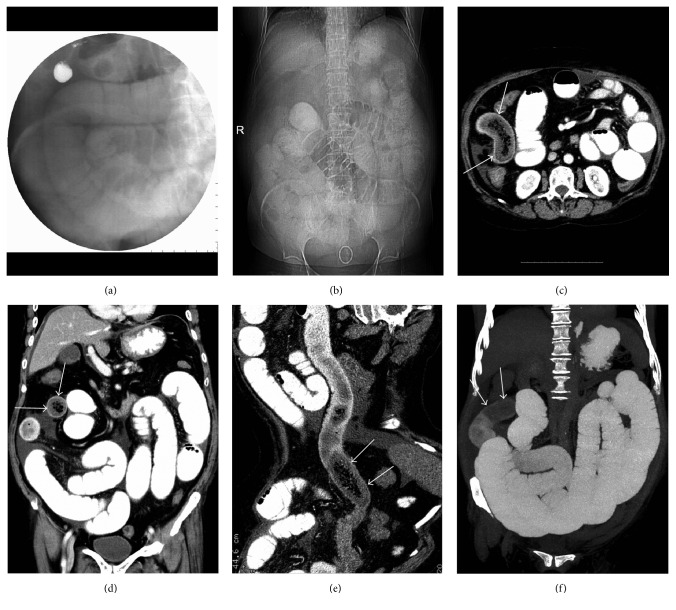
Phytobezoar in the middle segment of the small bowels causing SBO. (a) Gastrointestinal contrast radiography detected dilatation and fluid collection in the ileum. (b) Supine abdominal radiograph 24 hours after oral angiografin demonstration demonstrated no contrast agent accumulation in the colon which indicates a complete SBO. (c, d) MDCT axial and coronal images of the abdomen revealed a phytobezoar (white arrows) in the middle segment of the ileum and proximal distended bowels with contrast agent filling. (e) MDCT images with CPR clearly show the location of phytobezoar (arrows), proximal distended, and distal collapsed bowels. (f) MDCT with MIP image reveals a filling defect (arrows) at the obstruction site of the ileum and proximal distended small bowels.

**Figure 3:**
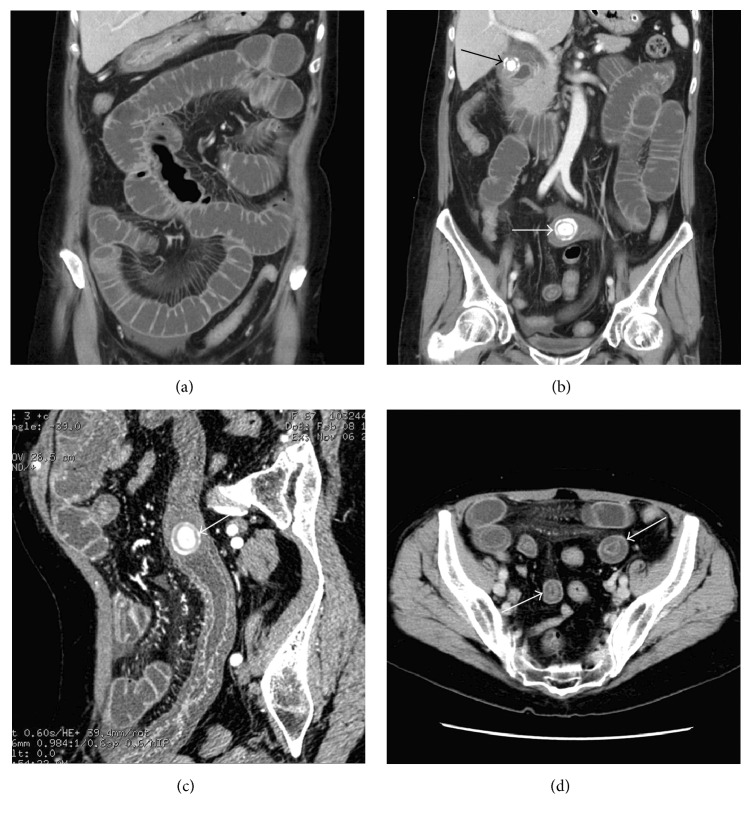
Calcareous bezoar in the distal segment of the small bowels causing SBO. (a) MDCT coronal image of anterior part of abdomen demonstrates small bowel dilatation and fluid accumulation. (b) MDCT coronal image of posterior part of the abdomen reveals a calcareous bezoar (white arrow) in the distal segment of the ileum and a gallstone (black arrow). (c) MDCT with CPR image clearly indicates the location of the calcareous bezoar (arrow), proximal bowel wall thickening and abnormal enhancement, mesenteric haziness and vascular engorgement, and a distal collapsed bowel. (d) MDCT axial image shows multiple target signs (arrows) in the small bowel proximal to the calcareous bezoar.

In the SBO group, the locations of the bezoars were in the proximal segment in 1 patient, which was caused by the trichobezoar that formed in the stomach and passed through the pylorus into the proximal jejunum (Figures 1(a)–1(c)); the middle segment in 10 patients (Figures 2(a)–2(f)); and the distal segment in 21 patients (Figures 3(a)–3(d)), respectively. The bezoars were trichobezoar in 1 patient (Figures 1(a)–1(c)), phytobezoars in 24 patients (Figures 2(c)–2(f)), and calcareous bezoars in 7 patients (Figures 3(b) and 3(c)), respectively. The shapes of the bezoars appeared as nodular or ovoid in 18 patients, short tubular in 11 patients, and multiple nodular or short tubular in 3 patients. The margins of all the bezoars were well defined. Their volumes ranged from 3.96 to 26.73 cm^3^ and CT values from −14 to 101 HU on the unenhanced images. Contrast-enhanced CT scans showed no enhancement in all the bezoars. Secondary changes were as follows: the bowel segment proximal to the bezoars was dilated in all 32 patients (mild, 13 patients; moderate, 15 patients; and severe, 4 patients); bowel wall thickening was observed in 22 patients; and vascular engorgement, mesenteric haziness, and ascites were found in 17, 11, and 6 patients, respectively. The contrast-enhanced CT scan showed moderate to high enhancement in the bowel wall proximal to the bezoars, and the degree of the enhancement in the portal venous phase was greater than that in the arterial phase. Other findings involved the presence of gallstones in 2 patients and kidney stones in 1 patient, respectively.

**Figure 4:**
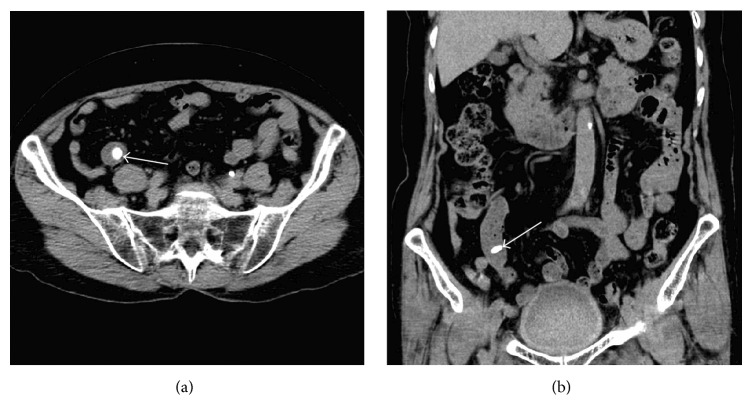
Calcareous bezoar in the distal segment of the small bowels without SBO. A nodular hyper dense calcareous bezoar (arrows) was found by accident in the axial (a) and coronal (b) images of the abdominal MDCT examination. The bezoar size was 1.1 × 1.0 × 0.7 cm and a volume of 0.403 cm^3^, but no any SBO sign or secondary change was observed in the patient.

In the non-SBO group, the locations of the bezoars were in the proximal segment in 5 patients, the middle segment in 11 patients, and the distal segment in 13 patients, respectively. This included the calcareous bezoars in 26 patients, which appeared as a round or ovoid hyper dense nodule with their CT values ranging from 80 to 335 HU (Figures 4(a) and 4(b)), and the phytobezoars in 3 patients. The major diameter of the bezoars ranged from 0.5 to 3.1 cm and their volumes from 0.065 to 9.328 cm^3^. These changes, including bowel dilatation, wall thickening, abnormal wall enhancement, target sign, vascular engorgement, and mesenteric haziness (Figures 4(a) and 4(b)) were not observed in the non-SBO group. In addition, 3 patients had gallstones, 3 had kidney stones, and 2 had pancreatitis.

The bezoars in the SBO group had greater mean values of both the major diameter and the volume and a lower value of the mean CT attenuation than those in the non-SBO group (*P* < 0.0001, resp., Table 2). Comparison between the two groups showed that the type of the bezoars (calcareous bezoar and phytobezoar) and the secondary bowel changes (including bowel dilatation, obstructive degree, wall thickening and abnormal enhancement) were significantly different (*P* < 0.0001). The target sign (*P* = 0.0001), mesenteric vascular engorgement (*P* < 0.0001) and haziness (*P* = 0.002) were also more commonly seen in the SBO group. Although the difference in the locations of the bezoars between the two groups was not significant, the bezoars in the SBO group were predominantly found in the distal ileum (Table 2).

In univariate logistic regression analysis, duration before admission, major diameter and volume of bezoars, and phytobezoar were possible risk factors associated with SBO, while CT value of bezoars and calcareous bezoar were possible protective factors (Table 3). The multivariate analysis showed that the major diameter of bezoar and phytobezoar remained as independent risk factors associated with the SBO (odds ratio = 36.09, 8.26, resp., and *P* = 0.0004, 0.044, resp., Table 4).

### 3.4. Treatment Results

In 15 of 32 patients with SBO, who received surgical intervention, various degrees of dilatation were seen in the small bowel proximal to the bezoars and small bowel ischemia in 5 patients. In one patient, who underwent gastroscopy and surgery, stomach and duodenum wall edema was observed. Opening the stomach showed a giant trichobezoar filling about 75% of its cavity, extending through the pylorus and the duodenum into the proximal jejunum, and was covered with viscous liquid secretions (Figure 1(d)).

The remaining 17 patients from the SBO group recovered uneventfully and were discharged following a conservative approach. All patients without SBO received either a conservative treatment or other treatments for bezoars or other diseases and recovered uneventfully. There were no recurrences of SBO episodes during 3–6 months of follow-up.

## 4. Discussion

Small bowel bezoars are divided into calcareous bezoars, phytobezoars, trichobezoars, foreign stuff, and bezoars from other sources, based on their contents. Calcareous bezoars are mainly gallstones, which are caused by the gallstones passing into the intestine [20]. Phytobezoars consist of undigested fiber, fruit seeds, or vegetable, which form and often remain in the stomach, and then migrate to the small bowel [18]. Trichobezoars are balls comprising swallowed hair and are mostly found in women younger than 30 years, who may be suffering from psychiatric disorders [23]. Other bezoars include foreign stuff and medicines, such as coins, buttons, screws, parts of toys, bubble gum, and candies, which are mainly found in children younger than 2 years [6].

In the present study, all patients with bezoars had one or more clinical symptoms, including abdominal pain, abdominal distension, constipation, nausea and vomiting, abdominal mass, and melena. Considering the clinical symptoms, although significant differences were observed between the two groups for the percentage of abdominal distension, constipation, and nausea and vomiting, these symptoms were not different from that in the SBO due to other causes. Diagnosis of SBO caused by bezoars was rarely established on the basis of the clinical findings before the MDCT era and requiring the surgical exploration [17, 23].

Radiological examinations are helpful in the diagnosis of SBO caused by bezoars. Though a preliminary location of the site of the SBO can be made from the abdominal radiograph findings, the cause and accurate location of SBO cannot be predicted. In 9 patients, digital fluoroscopy further confirmed the location and the degree of obstruction. However, the diagnostic value of the above-mentioned two radiological examinations is limited because the causes resulting in SBO are usually not apparent and relational complications are difficult to evaluate. These findings are similar to those described in previous reports [4].

It reveals that phytobezoar is the predominant type of bezoars causing SBO. Although there was no significant difference in the location of bezoars between the two groups, bezoars were more frequently located at the ileum (65.6%) in the SBO group. This reveals that the ileum may be the more susceptible site for SBO resulting from bezoars. It is believed that the location of the ileum at a lower position results in a delayed transit through the small bowel, with increased time for fluid absorption across the bowel wall. This leads to an accumulation of undigested food, as a result of stasis or obstruction. The terminal of ileum is more susceptible to bezoar-induced SBO probably due to its small diameter.

The bezoar with greater than 3 cm in diameter is easily impacted in the small bowel, especially the terminal ileum. The correlation between the size of the bezoars and incidence of SBO has been previously reported. Zissin et al. suggested that bezoars measuring approximately 3 × 5 cm may be regarded as a pathognomonic CT finding for an obstructing bezoar [16]. Kim et al. have also observed that the mean long-axis diameter of obstructing bezoars was 5.2 cm [15]. These reported measurements match the size of the surgically extracted bezoars documented in many case reports. We found that an increased diameter of bezoar was an independent predictive factor for SBO in patients with bezoar.

MDCT which provides multiplanar reconstructed image with thin slice sections has an advantage in diagnosis of the bezoars causing SBO. The images are able to characterize the types, size, and locations of the bezoars, the presence or absence of obstructions, the degree and level of obstruction, and relational complications. Thus, the radiologist may be able to make an overall evaluation of the patients' condition. It is beneficial to establish a proper treatment plan and effectively avoid unnecessary surgical intervention. Of the 32 patients with SBO in our study, only 15 patients underwent surgical intervention. The remaining 17 patients recovered with conservative treatments.

Typical phytobezoars appear as a well-defined, ovoid, or round intraluminal mottled-appearing mass containing air bubbles in the interstices [2–5, 16, 21]. A few phytobezoars also appear as a soft tissue mass without air, making diagnosis difficult, as it can resemble an intraluminal tumor [15]. Our study shows that all kinds of bezoars were not enhanced in the contrast-enhanced CT scanning that can differentiate between bezoars and tumors. The typical CT characteristics of calcareous bezoars are an ovoid, nodular or tubular, homogenous, or multilayer intraluminal hyper dense mass. In one patient, the trichobezoar was observed as a large, well-circumscribed, heterogeneous mass with entrapped air in the lumen of the stomach. The tail migrated from the stomach into the small bowel, appearing as concentric whorls of different densities. This rare type of trichobezoar is known as the Rapunzel syndrome [22, 24].

There are several limitations in the present study. First, the study is retrospective. Second, most patients without SBO did not undergo surgery or pathological evaluation to confirm the clinical and radiographic diagnosis. The diagnosis of SBO in the majority of the patients was not confirmed surgically. These patients had a clinical and radiographic diagnosis of SBO, which was confirmed on imaging.

In conclusion, we found that the bezoar causing SBO had more frequent phytobezoar type and larger volume measured on MDCT images and the major diameter of the bezoar is a possible risk factor for SBO. MDCT is a useful radiological technique for identifying the characteristics (types, size, locations, etc.) of bezoars, enabling a rapid and accurate etiological diagnosis, and in evaluating the degree of obstruction and secondary changes in SBO caused by bezoars.

## Figures and Tables

**Table 1:** Clinical characteristics and symptoms in the SBO and non-SBO groups.

Clinical findings	SBO group (*n* = 32)	Non-SBO group (*n* = 29)	*P* value
Clinical characteristics			
Age (years; mean ± SD)	56.69 ± 12.18	53.48 ± 12.12	0.308
Sex (M/F)	10/22	8/21	0.754
BMI (kg/m^2^; mean ± SD)	26.47 ± 4.26	25.83 ± 4.69	0.693
Symptoms [*n* (%)]			
Abdominal pain	32 (100)	26 (89.7)	0.203
Abdominal distension	15 (46.9)	0	<0.0001
Constipation	13 (40.6)	0	0.0004
Nausea and vomiting	10 (31.3)	0	0.003
Abdominal mass	4 (12.5)	0	0.147
Melena	0	3 (10.3)	0.203
Duration of symptoms before admission (*d* ± SD)	11.88 ± 7.61	7.76 ± 6.83	0.031

**Table 2:** MDCT findings in the bezoars, with and without small bowel obstruction.

MDCT findings	SBO group (*n *= 32)	Non-SBO group (*n *= 29)	*P* value
Bezoar features			
Major diameter (cm ± SD)	3.24 ± 0.53	1.62 ± 0.72	<0.0001
Volume (cm^3^ ± SD)	14.89 ± 6.41	2.52 ± 2.73	<0.0001
CT value (HU ± SD)	55.47 ± 23.39	173.03 ± 68.04	<0.0001
Location [*n* (%)]			
Proximal	1 (3.1)	5 (17.2)	0.128
Middle	10 (31.3)	11 (37.9)	0.781
Distal	21 (65.6)	13 (44.8)	0.169
Type [*n* (%)]			
Calcareous bezoar	7 (21.9)	26 (89.7)	<0.0001
Phytobezoar	24 (75.0)	3 (10.3)	<0.0001
Trichobezoar	1 (3.1)	0	1
Secondary changes			
Bowel changes			
Dilatation (cm ± SD)	3.26 ± 0.53	2.04 ± 0.26	<0.0001
Obstructive degree [*n* (%)]			
No	0	29 (100)	<0.0001
Mild	13 (40.6)	0	
Moderate	15 (46.9)	0	
Severe	4 (12.5)	0	
Wall thickening (mm ± SD)	3.56 ± 0.83	1.89 ± 0.40	<0.0001
Wall enhancement (HU ± SD)			
Unenhanced phase	35.69 ± 5.85	34.03 ± 6.40	0.296
Arterial phase^*∗*^	43.19 ± 15.06	28.93 ± 13.33	0.0001
Portal phase^*∗*^	65.06 ± 17.41	46.14 ± 11.50	<0.0001
Target sign [*n* (%)]	13 (40.6)	0	0.0001
Mesenteric changes [*n* (%)]			
Vascular engorgement	17 (53.1)	0	<0.0001
Mesenteric haziness	11 (34.4)	0	0.002
Ascites	6 (18.8)	3 (10.3)	0.355

^*∗*^The absolute difference in the attenuation value of the bowel wall between the unenhanced and contrast-enhanced images.

**Table 3:** Logistic regression analysis of risk factors influencing small bowel obstruction for patients with bezoars.

Variables	*B*	SE	Wald	*P* value	Odds ratio (95% CI)
Clinical characteristics					
Age	0.022	0.022	1.054	0.305	1.02 (0.98–1.07)
Sex	0.177	0.564	0.098	0.754	1.19 (0.40–3.60)
Duration before admission	0.082	0.040	4.317	0.038	1.09 (1.01–1.17)
Bezoar features					
Major diameter	3.835	1.025	14.006	0.0002	46.30 (6.21–345.03)
Volume	0.585	0.157	13.946	0.0002	1.80 (1.32–2.44)
CT value	−0.165	0.060	7.612	0.006	0.85 (0.75–0.95)
Location					
Proximal	−1.865	1.129	2.731	0.098	0.16 (0.02–1.42)
Middle	−0.296	0.540	0.300	0.584	0.74 (0.26–2.15)
Distal	0.854	0.527	2.626	0.105	2.35 (0.84–6.60)
Type					
Calcareous bezoar	−2.238	0.596	14.091	0.0002	0.11 (0.03–0.34)
Phytobezoar	2.064	0.582	12.552	0.0004	7.88 (2.51–24.66)

*B*: regression coefficient; SE: standard errors of regression coefficient.

**Table 4:** Multiple logistic regression analysis of risk factors influencing small bowel obstruction for patients with bezoars.

Variables	*B*	SE	Wald	*P* value	Odds ratio (95% CI)
Major diameter	3.586	1.016	12.470	0.0004	36.09 (4.93–264.13)
Phytobezoar	2.111	1.047	4.062	0.044	8.26 (1.06–64.31)

*B*: regression coefficient; SE: standard errors of regression coefficient.

## Abbreviations

SBO: Small bowel obstruction
CPR: Curved planar reformation
MPR: Multiplanar reformation
VR: Volume rendering
MIP: Maximum intensity projection.
